# Load–Velocity Relationship and 1RM Estimation of the Free-Weight Squat in Untrained Early-Adolescent Females

**DOI:** 10.3390/sports14020064

**Published:** 2026-02-05

**Authors:** Irene Sevilla-Arrabal, Diego A. Alonso-Aubin, Amador García-Ramos, Javier Courel-Ibáñez

**Affiliations:** 1 Department of Physical Education and Sports, Faculty of Sport Sciences, University of Granada, 18071 Granada, Spain; irenesevilla@correo.ugr.es (I.S.-A.); amagr@ugr.es (A.G.-R.); 2 Strength Training and Neuromuscular Performance Research Group (STreNgthP), Faculty of Health Sciences—HM Hospitals, University Camilo José Cela, C/Castillo de Alarcón, 49, Villanueva de la Cañada, 28692 Madrid, Spain; 3 HM Hospitals Health Research Institute, 28015 Madrid, Spain; 4 Department of Sports Sciences and Physical Conditioning, Faculty of Education, Universidad Católica de la Santísima Concepción, Concepción 2850, Chile

**Keywords:** adolescent, resistance training, muscle strength, development, basketball

## Abstract

**Background:** Velocity-based training (VBT) is used to estimate maximal strength and prescribe resistance-training loads, but evidence in untrained youth, especially early-adolescent females, is limited. In untrained early-adolescent females performing free-weight back squats, (1) the load–velocity relationship (LVR) is comparable to adult samples, albeit with greater between-subject variability, and (2) one-repetition maximum (1RM) estimates are affected by the minimum velocity threshold (MVT) anchor. **Methods:** Thirty-four untrained females (10–14 years) completed two progressive loading tests followed by actual 1RM attempts. Mean propulsive velocity (MPV) was recorded to model LVRs. Three MVTs were considered: (a) Actual (from Test 1), (b) General (0.30 m·s^−1^), and (c) Optimal (individualized to minimize prediction error in Test 1). LVR-based 1RM estimates from Multi-point and Two-point approaches were generated in Test 2 using each MVT and compared with the actual 1RM. **Results:** MPV decreased near-linearly with load (median R^2^ ≈ 0.996), from 1.00 ± 0.19 m·s^−1^ at ~40%1RM to 0.30 ± 0.05 m·s^−1^ at 100%1RM. Across MVTs, Two- and Multi-point models showed similar 1RM accuracy (≤~0.7% difference; *p* > 0.35). Actual and General MVTs overestimated 1RM (+5.1 kg; *p* < 0.001), whereas an individualized Optimal MVT (~0.38 m·s^−1^) removed bias (+0.6 kg; *p* = 0.52) and reduced error (*p* ≈ 0.03). **Conclusions:** In untrained early-adolescent females, the back-squat LVR is highly linear, and 1RM estimation accuracy hinges on the MVT anchor. A streamlined Two-point LVR paired with an individualized Optimal MVT provides an efficient, accurate workflow for youth strength assessment.

## 1. Introduction

The load–velocity relationship (LVR) enables practitioners to estimate relative intensity and maximal strength (1RM) from movement velocity, allowing load prescription and monitoring without repeated maximal attempts [1,2]. In adults, a near-linear association between mean barbell velocity and relative load (%1RM) has been shown consistently for the free-weight back squat, supporting velocity-based training (VBT) as an individualized framework for day-to-day load selection and submaximal 1RM estimation via the LVR [2,3].

Translating this approach to youth is not straightforward. Early adolescence is characterized by neuromuscular immaturity (e.g., less efficient motor-unit recruitment and inter/intra-muscular coordination) and limited technical proficiency, factors that can increase velocity variability and alter LVR [4,5]. Moreover, biological maturation influences strength–velocity characteristics, suggesting that adult-derived equations may misestimate 1RM or relative intensity in developing athletes [6,7]. Despite growing interest in VBT within youth programs [8,9], reference velocity values and validated LVR-based 1RM estimates remain scarce for untrained adolescents, especially for girls, who are underrepresented in the literature.

Although properly supervised 1RM testing can be safe for children and adolescents, and is endorsed when conducted under appropriate procedures [10,11], logistical constraints in real-world settings (large groups, limited supervision time, and variable technique) often reduce feasibility. Submaximal, velocity-informed methods are therefore attractive, but their accuracy depends critically on the choice of the minimum velocity threshold (MVT) used to anchor the prediction [3].

Accumulating evidence in trained adults indicates that bias is primarily driven by MVT selection rather than the specific regression form. Individualized or “optimal” MVTs tend to outperform fixed/group values, and when the anchor is held constant, Two-point and Multi-point models yield comparable 1RM accuracy [12,13,14,15]. Whether these findings extend to untrained early-adolescent females is unknown.

Therefore, to inform practical VBT load guidance in untrained youth, this study aimed to (i) characterize the general relationship between relative load (%1RM) and mean propulsive velocity (MPV) in the free-weight parallel back squat of untrained early-adolescent females, and (ii) compare 1RM estimates modelled by LVR using Multi-point and Two-point approaches combined with three MVTs.

We hypothesized that the MPV–%1RM profile would be inverse and approximately linear across 40–100% 1RM in our untrained youth sample, consistent with adult free-weight data in women, albeit with greater between-subject variability than trained adults [1,16,17]. We further hypothesized that, when the velocity anchor is held constant, Two-point and Multi-point models would show comparable 1RM estimation accuracy, as suggested by women’s 2–3 point work [15,18]. Finally, we expected an individualized Optimal MVT (derived independently at T1 and applied to T2) to remove group-level bias and reduce absolute errors compared to Actual or General anchors [12] extending women-specific evidence that anchor choice, rather than model form, governs prediction accuracy [15,18].

## 2. Materials and Methods

### 2.1. Study Design

This was a descriptive laboratory study using a within-subject repeated-measures design to examine the LVR in the free-weight back squat in early female adolescents and compared the 1RM prediction accuracy of two common regression models (Multi-point and Two-point) combined with three minimum velocity threshold (MVT) procedures: Actual MVT, General MVT, and Optimal MVT. After a familiarization session, participants completed two progressive loading tests (T1 and T2) on separate days (48–72 h). All sessions took place at the same time of day (±1 h) in similar environmental conditions (28–29 °C, 52–56% humidity), using the same equipment and footwear. Participants avoided strenuous lower-body exercise for ≥48 h before testing, maintained normal hydration, and refrained from caffeine for 3–4 h. T1 established each athlete’s individual LVR via a progressive loading test up to 1RM during the squat and was used to determine Actual MVT (observed MPV at 1RM) and Optimal MVT (derived from the individual LVR); General MVT was set to 0.30 m·s^−1^ according to most conventional standards [2,12,19]. T2 repeated the progressive loading test and provided the dataset for model fitting and comparisons: the Multi-point model used all eligible loads, and the Two-point model used a light–heavy pair extracted from the same session. The actual 1RM reached at T2 was estimated using the LVR from T2 combined with the MVTs defined at T1. Direct 1RM testing was required to provide a criterion reference against which the accuracy of indirect, velocity-based 1RM estimates could be validly evaluated.

### 2.2. Participants

Untrained early-adolescent females aged 10 to 14 years were recruited from a local female basketball club. Inclusion criteria were (1) no prior resistance-training experience; (2) ability to follow instructions; (3) no physical limitations affecting testing; and (4) no serious injury in the previous 6 months. Written informed consent was obtained from participants and their parents/legal guardians before testing. The protocol was approved by the local Ethics Committee (REF: 4222/CEIH/2024). Biological maturation status (e.g., Tanner stage or estimated peak height velocity) was not assessed. A priori, we powered the study to (i) detect at least a large correlation in the within-subject load–velocity relationship (*r* = 0.50) with 80% power at α = 0.05 (*n* = 29) [20], and (ii) detect a paired difference of 2.5 kg in 1RM estimation bias between methods, assuming a standard deviation of the differences, SDs = 5 kg [21], which required *n* = 32.

### 2.3. Measurement and Equipment

Height was assessed to the nearest 0.5 cm using a stadiometer (SECA 217, Berlin, Germany). Body weight was measured using a segmental body composition scale (TANITA BC 545N, Innerscan, Tokyo, Japan). A free-weight rack, a 5 kg Olympic barbell, and calibrated weight discs (PowerKan, Valladolid, Spain) were used for the assessment. Barbell kinematics (velocity and range of motion) were recorded in real time with a valid linear position transducer (Chronojump, BoscoSystem, Barcelona, Spain), placed on the ground to the right of the participants’ feet, with the Velcro strap attached 0.50 m to the right of the barbell center. Mean propulsive velocity (MPV) was selected as the primary velocity outcome to mitigate braking-phase artefacts and closely tracks %1RM in the squat [22].

### 2.4. Back-Squat Exercise Description

Given participants’ limited training history, we used a parallel squat standard to balance safety, feasibility, and measurement reliability [23]. Participants performed the parallel free-weight back squat using a high-bar position (barbell resting across the upper back/shoulders). They stood upright with hips and knees fully extended and feet approximately shoulder-width apart. On a verbal cue, they initiated a controlled descent (~1.2 s) to a standardized depth using guide bands until reaching parallel depth, defined as the point where the femur was parallel to the floor [23]. Without pausing, they immediately ascended with maximal intent, using the stretch–shortening cycle. Real-time auditory feedback of MPV was provided after each repetition. To minimize velocity fluctuations, participants were instructed to keep the barbell in continuous contact with the shoulders, avoid leaving the ground at lockout, and refrain from using weightlifting belts.

### 2.5. Progressive Loading Test

A standardized warm-up (2 sets of 5 repetitions of the back squat, against 5 and 10–15 kg) preceded each test. In both sessions, loads were adjusted to reach four target loads: Light (~30% RM; ~1.00 m·s^−1^), Moderate (~55% RM; ~0.80 m·s^−1^), Heavy (~80% RM; ~0.60 m·s^−1^), and actual 1RM. Light and Moderate loads were confirmed with 2–3 repetitions; Heavy and 1RM loads with one repetition. Interset rest was standardized (~2 min after Light, 3 min after Moderate, 4 min after Heavy/1RM) to control fatigue and preserve repetition velocity [24]. For each load, the fastest valid repetition (highest MPV) was retained for modelling the L–V regression.

### 2.6. General Load–Velocity Modelling

At T2, each participant’s LVR was modelled with a single individualized linear regression: load (kg) = a + b·MPV, fitted with the fastest valid repetition at Light, Moderate, Heavy, and the 1RM trial. From the fitted line, a 40–100% 1RM grid in 5% increments was generated by evaluating at kg target = (% × 1RM) to obtain reference MPVs for each %1RM. The reference MPVs at each 5%1RM were then averaged across participants to obtain a general %1RM–MPV relationship.

### 2.7. 1RM Estimation

To estimate 1RM at T2 without circularity (i.e., independence between anchor derivation and prediction), we fit submaximal L–V models that excluded the 1RM point under two approaches: (1) Multi-point and (2) Two-point. For each approach, we applied three velocity anchors (MVTs) determined from T1: [12] (1) Actual MVT (the individual MPV recorded at the actual 1RM in T1); (2) General MVT (0.30 m·s^−1^ for all), and (3) Optimal MVT (the individual velocity that minimizes the difference between actual and predicted 1RM, computed as Optimal MVT = [actual 1RM load − intercept]/slope) [25]. Each MVT anchor was then entered into the two submaximal L–V equations to yield six predictions per participant.

### 2.8. Statistical Analyses

For each %1RM on the 40–100% grid, reference MPVs were summarized across participants as mean ± SD with 95% CIs and between-subject coefficient of variation (CV % = 100·SD/mean). Individual goodness-of-fit was expressed as *R*^2^ per participant and reported as median and range. The 1RM prediction accuracy was defined as raw and absolute errors. Within-model bias was tested with paired *t*-tests on the raw error, and we report bias ± SD and 95% limits of agreement (LoA = bias ± 1.96·SD). Between-model comparisons were tested using a linear mixed-effects model with approach (Multi- vs. Two-point), MVT (Actual, General, Optimal), and their interaction as fixed effects, and a random intercept for participant. The dependent variable was absolute error (kg). The same model was re-run on raw error (kg) to examine the direction of bias. Estimated marginal means with Holm adjustment were used for pairwise contrasts. Model assumptions were checked on model residuals. Participants were classified as “low-error” (<5%) and “moderate-error” (5–10%) responders based on absolute percentage error (APE (%) = 100 × |Pred − Actual|/Actual) [13]. Changes in responder proportions between MVT procedures were tested with McNemar’s test. All analyses were conducted in RStudio (v.2024.12.1). Statistical significance was set at *α* = 0.05.

## 3. Results

Thirty-four untrained early adolescents completed the tests (Table 1). Participants lifted 8.4 ± 1.4 loads and 17.3 ± 3.3 repetitions to complete T1 and 8.4 ± 1.6 loads and 15.6 ± 3.8 repetitions to complete T2 (Table 2).

In T2, participants lifted heavier loads at Light (Mean difference: 1.3 kg, *p* = 0.008, *d* = 0.48), Moderate (Mean difference: 2.0 kg, *p* = 0.003, *d* = 0.57), and Heavy (Mean difference: 2.7 kg, *p* = 0.001, *d* = 0.61) attempts and reached faster velocities at Light attempts (Mean difference: +0.066 m·s^−1^, *p* = 0.048, *d* = 0.35). The 1RM was not significantly different between sessions. These between-session increases, particularly at lighter loads, likely reflect learning or familiarization effects, which are expected in untrained early-adolescent populations.

Table 3 shows the reference mean propulsive velocities (MPV) estimated from the complete Multi-point model in T2. Across the 40–100% 1RM range, MPV decreased almost linearly from 1.00 ± 0.19 m·s^−1^ at 40% 1RM to 0.28 ± 0.05 m·s^−1^ at 100% 1RM. The between-subject variability remained moderate to large across loads (SD = 0.05–0.19 m·s^−1^; CV 11.3–18.7%), consistent with the heterogeneity of the early-adolescent cohort. Individual fits of the final multi-point model were excellent (median *R*^2^ = 0.996, range 0.943–1.000).

**Figure 1:**
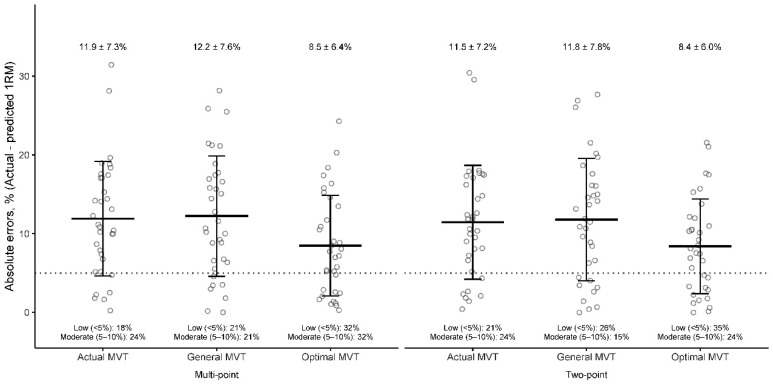
Comparison of the absolute errors between the actual and predicted one-repetition maximum (1RM) estimated by two types of load–velocity profiling models (Multiple-point and Two-point) using three types of minimum velocity thresholds (MVTs: actual MVT, general MVT, and optimal MVT) during the free-weight back squat in untrained early-adolescent females. Bars represent absolute prediction error (kg) between actual and estimated 1RM. Means ± SDs are indicated. 1RM, one-repetition maximum.

Optimal MVT was virtually identical between Multi- and Two-point models (0.343–0.407 vs. 0.344–0.406 m·s^−1^; *p* = 0.821) and clearly higher than Actual MVT (0.276–0.312 m·s^−1^; mean Δ = 0.081 m·s^−1^; *p* < 0.001, *d* = 1.08). Pairwise comparisons showed that using the Optimal MVT improved 1RM prediction accuracy versus Actual and General MVT in both LVR approaches: in Multi-point, Δerror = −3.99% vs. Actual (*p* = 0.032, *d* = 0.44) and −5.65% vs. General (*p* = 0.032, *d* = 0.49); in Two-point, Δerror = −3.62% vs. Actual (*p* = 0.032, *d* = 0.40) and −6.10% vs. General (*p* = 0.032, *d* = 0.42). Holding the MVT procedure constant, Two-point vs. Multi-point regressions showed negligible differences (Δerror = −0.71% to +0.11%; all *p* = 0.355–0.691). The McNemar analyses indicated that Optimal MVT increased the proportion of “low-error” responders by +8.8 to +14.7% across approaches, but these changes were not statistically significant (*p* = 0.267–0.546). For “moderate-error” responders, improvements were +0.0 to +11.8%, also non-significant (*p* = 0.423–1.000). Model–error distributions are shown in Figure 1.

Using Actual or General MVT produced a clear positive bias in the 1RM estimates (Table 4) in both LVR approaches (Pred−Actual ≈ +5.1 to +5.2 kg; all *p* < 0.001; *d* = 0.89–1.04). In contrast, with the Optimal MVT, the bias was essentially removed (+0.6 ± 5.4–5.5 kg), with no significant difference from the actual 1RM (*p* = 0.515–0.546; *d* = 0.10–0.11). Results were virtually identical for Multi-point and Two-point profiling (e.g., Predicted 1RM: 58.2–58.4 kg for Actual/General vs. 53.7–53.8 kg for Optimal).

## 4. Discussion

This study provides reference MPV values for practical load guidance and monitoring of the free-weight back squat in untrained early-adolescent females. The MPV–%1RM relationship showed the expected smooth, monotonic decline from 40% to 100% 1RM, indicating sound execution and measurement across the loading spectrum, with high individual fit. Between-subject dispersion was moderate at mid-loads and increased toward 1RM, consistent with an untrained cohort and aligned closely with adult free-weight profiles under comparable specifications. For 1RM estimation, the individualized Optimal MVT provided unbiased point estimates at the group level, whereas Actual and General anchors produced a systematic positive bias.

Nonetheless, error distributions remained wide, and a non-trivial subset of participants fell outside training-useful bounds on absolute error. Thus, while an Optimal anchor improves average accuracy, practical uncertainty at the individual level persists and should be considered in untrained youth monitoring and load guidance. It is important to emphasize that the Optimal MVT represents a statistical, error-minimizing anchor derived retrospectively from the load–velocity relationship, rather than a physiological, biomechanical, or neuromuscular threshold. Therefore, it should not be interpreted as a stable individual characteristic, but as a modelling parameter dependent on the testing protocol and population. Furthermore, sex- and maturation-specific anchoring values remain necessary and should not be assumed interchangeable.

Our LVR reference (MPV–%1RM) in untrained early-adolescent females followed a smooth decline from ~1.00 m·s^−1^ at 40% 1RM to ~0.28 m·s^−1^ at 100%, aligning closely with adult free-weight profiles under comparable specifications. In trained adult women [1], MPV anchors across 40–100% 1RM are similar, consistent with the expectation that a greater range of motion (ROM; full vs. parallel squat) yields lower velocities at a given relative load. In contrast, recreational young women performing the full squat exhibit lower MPV values at heavier loads, reflecting lower training status [1]. Studies reporting mean velocity (MV) in trained women [16,26] also confirm a linear LVR and show slightly higher per-% velocities (e.g., ~1.05–1.12 m·s^−1^ at 40%, ~0.70–0.74 m·s^−1^ at 70%, ~0.35 m·s^−1^ at 100% in the full squat), consistent with metric/device implementation rather than physiology [16]. Because most female datasets rely on the full back squat, they are not a perfect match for our parallel-squat protocol; the close agreement observed at moderate loads (40–70% 1RM) likely reflects opposing influences of ROM and training status, resulting in similar anchors.

In the absence of women-specific parallel-squat datasets, our values should be interpreted as parallel-squat references. Cross-ROM pooling, particularly at 1RM, should be avoided, and remaining heavy-end discrepancies are best attributed to ROM, metric/device implementation, and reliability limits near maximal loads. As anticipated, between-subject SD at 40% 1RM was higher in our untrained cohort (0.19 m·s^−1^) than in trained-women reports (0.10–0.12 m·s^−1^), but comparable at 70% 1RM (0.09 vs. 0.07 m·s^−1^) [1,16]. Relative dispersion (CV%) was greater in our cohort at 40–70% (18.7% and 13.9% vs. ~9–11% in trained women) and highest in recreational women at heavy loads (70%: 17.4%; 100%: 26.1%) [1,16,17]. At 100% 1RM, SDs were similar across cohorts (~0.05–0.06 m·s^−1^), but CVs inflated in all groups (ours: 16.6%; trained MV: 14.3%; trained MPV: 20.0%; recreational: 26.1%), consistent with lower velocity reliability near maximal loads [1,16,17]. Collectively, training status/technical proficiency, execution/depth standardization, and lower reliability near 1RM account for the heavy-end spread, while moderate loads ~70% convergence supports the external validity of our reference anchors.

Although direct evidence of LVR in adolescent populations is limited, our findings in untrained early-adolescent females are conceptually consistent with reports in trained youth, where squat-based load–velocity relationships are systematic and highly linear, yet context-dependent and influenced by training status and methodological choices [9]. From a practical perspective, MVTs in untrained youth should be considered dynamic rather than fixed. Periodic recalibration following familiarization, technical improvement, or strength gains is recommended, and velocity-based outputs should be integrated with technical assessment, perceived exertion, and training objectives rather than used in isolation.

Both Multi- and Two-point models showed comparable 1RM accuracy when holding the MVT constant, underscoring that the anchor and not the number of loads drives prediction error. In our cohort, the individualized Optimal MVT ~0.375 m·s^−1^ yielded unbiased 1RM estimates and reduced absolute error, whereas Actual (~0.28–0.31 m·s^−1^) and a General 0.30 m·s^−1^ anchor produced systematic positive bias. Prior women-specific evidence in the free-weight full squat agreed that accuracy hinges on the anchor. In trained adult women [15], a sample-based fixed MVT of 0.25 m·s^−1^ produced no difference vs. measured 1RM, with SEE ≈ 1.21–1.76 kg, whereas 0.30 m·s^−1^ and 0.40 m·s^−1^ MVT underestimated 1RM and inflated error (SEE ≈ 4.20–4.26 kg and ≈12.6 kg, respectively). Similarly, in trained young adult females performing full back squats with MV [18], an individual (measured) MVT obtained within the same session yielded absolute differences of ~4.9–9.7 kg across Multi- vs. Two-point methods, with no significant method effect and ~3 kg more precise than the group MVT.

Notably, these studies did not implement a participant-specific Optimal MVT to minimize cross-session error. In contrast, our untrained early-adolescent cohort (parallel squat, MPV) applied a participant-level Optimal MVT derived explicitly to minimize T2 prediction error (with T1 anchors to avoid same-session bias), which removed group bias and lowered absolute percentage error versus Actual/General anchors across both model forms. Nevertheless, individual error dispersion remained wide (non-significant increases in <5% “low-error” responders), reinforcing that anchors must be sex- and protocol-specific. For youth parallel-squat load guidance, individualized Optimal anchors are preferable in practice, and if a fixed value is unavoidable, ~0.375 m·s^−1^ (MPV) is a defensible, data-driven starting point to verify and periodically recalibrate with heavy sets. This value should be considered a provisional, population- and protocol-specific reference derived from untrained early-adolescent females performing the parallel squat, rather than a broadly validated benchmark.

The results of this study should be interpreted with caution, considering that (i) the sample comprised untrained early-adolescent females from a single sport context performing the free-weight back squat, which constrains generalizability to trained adolescents, boys, or alternative squat styles; (ii) learning and familiarization effects across sessions may have subtly influenced LVR characteristics, particularly at lighter loads, although this does not affect the primary comparison of MVT anchoring strategies; and (iii) unmeasured differences in biological maturation may partly explain inter-individual variability in LVR characteristics and MVT values.

This study is strengthened by being the first to investigate the load–velocity relationship in free-weight exercises for untrained early-adolescent females with no prior weight training experience. Barbell kinematics were captured with a valid linear position transducer and standardized depth and rest intervals, strengthening internal validity. Analyses were conducted with rigorous within-subject comparisons and confidence intervals across finely spaced load bins, enhancing interpretability for practice. Fixing minimum velocity thresholds (MVTs) from an independent session (T1) and applying them to a subsequent session (T2) has been recommended to reduce same-session coupling between anchor derivation and prediction, thereby allowing clearer isolation of anchor-related effects on 1RM estimation accuracy [12,13,25]. Consistent with this rationale, the present results showed that systematic bias in 1RM estimation was driven primarily by the choice of the velocity anchor rather than by the regression approach itself, as Two-point and Multi-point models yielded comparable accuracy when the MVT was held constant. Although the Two-point method in this study was derived post hoc from a Multi-point loading protocol, the use of only one additional submaximal load between the Light and Heavy conditions limited fatigue-related velocity drift [24] and closely reflected streamlined Two-point testing procedures commonly applied under field-based velocity-based training conditions [2,19]. The criterion validation of prediction models required a true 1RM reference, conducted under strict supervision, progressive loading, and established international safety guidelines for youth resistance training. Because the sample consisted of untrained early-adolescent females from a single sport context, the observed variability patterns and Optimal MVT values may not generalize to males, trained youth, or adolescents at different stages of biological development.

## 5. Conclusions

The free-weight parallel back squat MPV–%1RM relationship in untrained early-adolescent females shows a smooth, near-linear decline across 40–100% 1RM with excellent individual fits. The choice of MVT anchor is the primary determinant of 1RM prediction accuracy: Actual (≈0.28–0.31 m·s^−1^) and General (0.30 m·s^−1^) anchors introduce systematic positive bias, whereas an individualized Optimal MVT (~0.375 m·s^−1^) removes bias and reduces absolute error. This reference value should be considered provisional and population-specific, and may shift with training status, biological maturation, squat depth, or technical proficiency; therefore, it should be verified and periodically recalibrated when applied in other youth populations or across training phases. When the anchor is held constant, Two-point and Multi-point LVRs perform similarly, supporting efficient Two-point profiling in practice. Because individual errors remain substantial, LVR-based 1RM estimates in untrained youth should be used primarily for relative load guidance and monitoring rather than for precise load prescription.

## Figures and Tables

**Table 1:** Sample characteristics.

Variable	Value
Sample (n)	34
Age (y)	12.3 ± 1.1
Height (cm)	158.0 ± 6.8
Weight (kg)	53.2 ± 10.5
1RM (kg)	53.1 ± 10.1
1RM/BM (ratio)	0.95 ± 0.17

1RM: One-repetition maximum. BM: Body mass; Data are means ± SD.

**Table 2:** Characteristics of the testing sessions.

		Light	Moderate	Heavy	1RM
T1	Load (kg)	15.4 ± 5.2 *	27.5 ± 7.2 *	39.2 ± 8.8 *	49.8 ± 9.4
	MPV (m·s^−1^)	0.99 ± 0.10 *	0.78 ± 0.08	0.56 ± 0.07	0.29 ± 0.05
	Relative load (%1RM)	30.2 ± 5.8	55.0 ± 7.6	78.5 ± 5.7	100
T2	Load (kg)	16.8 ± 6.0 *	29.5 ± 7.8 *	41.9 ± 9.2 *	53.1 ± 10.1
	MPV (m·s^−1^)	1.05 ± 0.17 *	0.81 ± 0.04	0.59 ± 0.06	0.28 ± 0.05
	Relative load (%1RM)	31.3 ± 7.6	55.2 ± 8.6	78.6 ± 5.7	100

T1: Session 1; T2: Session 2; 1RM: One-repetition maximum; MPV: Mean propulsive velocity. Data are presented as means ± SD. * Between-session differences, paired sample *t*-tests, *p* < 0.05.

**Table 3:** Mean propulsive velocity (MPV) related to each relative intensity (%1RM) for the free-weight back squat in untrained early-adolescent females.

%1RM	MPV (m·s^−1^)	95% CI	CV (%)
40%	1.00 ± 0.19	0.93–1.06	18.7
45%	0.94 ± 0.17	0.88–1.00	18.0
50%	0.88 ± 0.15	0.83–0.93	17.4
55%	0.82 ± 0.14	0.77–0.87	16.6
60%	0.76 ± 0.12	0.72–0.81	15.8
65%	0.71 ± 0.11	0.67–0.74	14.9
70%	0.65 ± 0.09	0.62–0.68	13.9
75%	0.59 ± 0.08	0.56–0.62	12.9
80%	0.53 ± 0.06	0.51–0.56	12.0
85%	0.48 ± 0.05	0.46–0.49	11.3
90%	0.42 ± 0.05	0.40–0.44	11.5
95%	0.36 ± 0.05	0.34–0.38	13.4
100%	0.28 ± 0.05	0.26–0.30	18.7

1RM: One-repetition maximum; MPV: Mean propulsive velocity; CV: Coefficient of variation.

**Table 4:** Paired sample *t*-tests comparing the actual 1RM and the 1RM estimated by the two LVR (Multiple-point and Two-point) and the three types of MVTs (Actual MVT, General MVT, and Optimal MVT).

LVR	MVT	Predicted 1RM (kg)	Raw Errors (kg)	*p*-Value	ES
Multi-point	Actual MVT	58.4 ± 11.9	5.2 ± 5.3	<0.001	0.99
	General MVT	58.2 ± 11.9	5.1 ± 5.7	<0.001	0.89
	Optimal MVT	53.8 ± 9.9	0.6 ± 5.5	0.515	0.11
Two-point	Actual MVT	58.4 ± 11.8	5.2 ± 5.0	<0.001	1.04
	General MVT	58.2 ± 11.9	5.1 ± 5.5	<0.001	0.92
	Optimal MVT	53.7 ± 10.0	0.6 ± 5.4	0.546	0.10

LVR: Load–Velocity relationship; 1RM: One-repetition maximum; ES: Cohen’s d effect size; MVT: minimum velocity threshold; *p*-value obtained from paired *t*-tests. Data are means ± SD.

## Data Availability

The data are not publicly available due to privacy or ethical restrictions. The data that support the findings of this study are available on request from the corresponding authors.
